# Fermentation of Apple Juice with a Selected Yeast Strain Isolated from the Fermented Foods of Himalayan Regions and Its Organoleptic Properties

**DOI:** 10.3389/fmicb.2016.01012

**Published:** 2016-06-28

**Authors:** S. S. Kanwar, Keshani

**Affiliations:** Department of Microbiology, CSK Himachal Pradesh Agricultural UniversityPalampur, India

**Keywords:** Western Himalayas, fermented foods, *Saccharomyces cerevisiae*, *ATF1* gene, apple cider

## Abstract

Twenty-three *Saccharomyces cerevisiae* strains isolated from different fermented foods of Western Himalayas have been studied for strain level and functional diversity in our department. Among these 23 strains, 10 *S. cerevisiae* strains on the basis of variation in their brewing traits were selected to study their organoleptic effect at gene level by targeting *ATF1* gene, which is responsible for ester synthesis during fermentation. Significant variation was observed in *ATF1* gene sequences, suggesting differences in aroma and flavor of their brewing products. Apple is a predominant fruit in Himachal Pradesh and apple cider is one of the most popular drinks all around the world hence, it was chosen for sensory evaluation of six selected yeast strains. Organoleptic studies and sensory analysis suggested Sc21 and Sc01 as best indigenous strains for soft and hard cider, respectively, indicating their potential in enriching the local products with enhanced quality.

## Introduction

Fermented food products are essential component of diet in a number of developing countries and are more common among people belonging to the rural areas, especially in hilly and tribal people, where the limited resources encourage the use of these products for the fulfillment of additional nutritional requirements (Kanwar et al., 2007). The knowhow of these traditional processes and technologies involved in the production of fermented products is being transferred from generation to generation as trade secrets. These fermented foods are made under primitive conditions, which result in low yield and poor quality and sometimes even in spoilage of the product. So there is a need to select the specific microflora associated with these products to maintain consistency in their production and quality. The most important organism associated with fermented food products is yeast and it has been observed that among several yeasts, *Saccharomyces cerevisiae* is the most common species associated with fermentation processes (Querol and Fleet, 2006). To preserve the typical organoleptic properties of the fermented product or beverage, it is essential to select a particular strain of yeast that imparts characteristic sensory and aromatic flavor to fermented product/beverage. Production of several wines from some tropical fruits using *S. cerevisiae* strains has already been reported (Ezeronye, 2004; Capece et al., 2012). Apple is one of the prominent fruit of Western Himalayas and is highly perishable. Hence, it is required to be processed to preserve its nutritive value and to develop value added products.

Western Himalayan region is a rich repository of microbial genetic diversity. Forty-three indigenous isolates of yeasts had already been characterized in the Department of Microbiology, Himachal Pradesh Agricultural University, Palampur from various fermented foods of Western Himalayas. Twenty-three of them were identified as strains of *S. cerevisiae* by conventional and molecular marker techniques such as Randomly Amplified Polymorphic DNA (RAPD), Inter Simple Sequence Repeats (ISSR), Universal Rice Primers (URP), and Delta markers (Pathania et al., 2010). These strains have already been studied for strain level diversity using internal transcribed spacer (ITS) region as a marker (Keshani et al., 2015). Further, on the basis of variation in brewing traits of these strains; they were further studied for their organoleptic effect at gene level. During fermentation processes, yeast cells produce a broad range of aroma-active substances especially volatile esters which greatly affect the complex flavor of fermented alcoholic beverages. While these secondary metabolites are often formed only in trace amounts, their concentration determines the distinct aroma of these beverages. The best-known enzymes involved in ester synthesis are alcohol acetyltransferases (AATases; EC 2.3.1.84). These AATases are encoded by *ATF1*, the *ATF1* homolog Lg-*ATF1*, and *ATF2* genes (Fujii et al., 1996; Yoshimoto et al., 1998; Yoshimoto et al., 1999). Verstrepen et al. (2003) demonstrated that overexpression of *ATF1* in a commercial brewer’s strain led to significant increase in concentrations of isoamyl acetate and ethyl acetate in the product. These results indicate that the expression level of *ATF1* is an important limiting factor for ester synthesis under industrial conditions. The variation in *ATF1* gene could also be revealed by organoleptic studies and then comparing the profiles with variations observed at genetic level. This study will further help in comparison of the ester profiles encoded by *ATF1* gene sequence, of the selected strains for understanding and determining the range of flavor phenotypes (esters) that wine yeasts of Western Himalayas exhibit, and how this knowledge can been used to develop novel flavor-active yeasts or to incorporate these wild yeasts with great fermentation (flavor) potential in industrial sector for better utilization at commercial level.

## Materials and Methods

### Yeast Isolates and Culture Maintenance

Out of 23 strains of *S. cerevisiae* available in the Department of Microbiology, HPAU, Palampur, India, 10 strains were used in the present investigation on the basis of variation in their brewing traits (**Table 1**) and were maintained on potato dextrose agar at 4°C and in 50% (v/v) glycerol at –80°C.

**Table 1:** *Saccharomyces cerevisiae* strains used in the present investigation along with their source, place of collection, and GenBank accession numbers of *ATF1* gene.

S. No.	Strain code	Source	Place of collection	GenBank^a^ accession number
1	Sc01	*Chhang*	Lahaul & Spiti	KF429732
2	Sc03	*Dhaeli*	Lahaul & Spiti	KF429733
3	Sc04	*Aara*	Lahaul & Spiti	KF429730
4	Sc05	*Chiang*	Lahaul & Spiti	KF429734
5	Sc 11	*Chuli*	Sangla	KF429737
6	Sc 12	Apple wine	Sangla	KF429739
7	Sc 15	Beverage	Bharmour	KF429736
8	Sc 19	Wine	Sangla	KF429735
9	Sc 21	Wine	Sangla	KF429738
10	Sc 24	Fermented product	Palampur	KF429731

*^a^GenBank, National Centre for Biotechnology Information (NCBI), USA.*

### *ATF1* Gene Studies

For DNA isolation, Yeast DNA isolation Kit was used (Biobasic Inc.). The DNA stock samples were quantified using Nanodrop. Quality and purity of DNA were checked by 0.8% agarose gel electrophoresis. For *ATF1* gene sequence, 293bp of upstream related to promoter and TATA box followed by 1578 bp of ORF and 217 bp of 3′UTR was used. For amplifi cation and sequencing, this 2088 bp region was divided into three overlapping sequences. Three separate primer pairs were used to amplify these three overlapping sequences, i.e., ATF1FL (TGCACTCGATGGTCTTCTCA) and ATF1FR (GACAAATTAGCCGCCAACTC) for the first contig, ATF1SL (TGCAATGTTCTGCACGTTATT) and ATF1SR (TAGTTGTGAGCGGCAATCTG) for the second contig and ATF1TL (GAACTTCGAATGGCTTACGG) and ATF1TR (TGCAATGTTCTGCACGTTATT) for the third contig. Polimerase chain reaction (PCR) amplification was carried out in the thermal cycler (BOECO, Germany) with an initial denaturation at 95°C for 2 min, followed by 30 cycles of 94°C for 30 s, 51°C for 30 s, and 72°C for 90 s with a final elongation step at 72°C for 10 min. The PCR product was analyzed on 1.2% agarose gel. For DNA sequencing, purified PCR products were freeze dried (CHRIST ALPHA I-2LD) and custom sequenced (ABI 3730xl automated sequencer) with both forward and reverse primers (Xcelris Labs Ltd., Ahmedabad, India). The overlapping regions of DNA sequences were aligned for retrieving complete gene sequence. The homology search for *ATF1* gene was carried out using NCBI BLASTN program http://www.ncbi.nih.gov/blast and phylogenetic analyses were conducted in MEGA 5.1 software program.

### Organoleptic Studies

Royal Delicious apple variety was selected for conducting experiments. Healthy fruits were selected, washed in hot water, mixed with 0.1% of potassium metabisulphite and then used for the extraction of juice under hygienic conditions. The physico-chemical analysis of apple juice was carried out for different parameters which included estimation of total soluble solids (TSS), pH, titrable acidity, brix acid ratio, total sugars, reducing sugars, and ascorbic acid. Starter culture of six selected *S. cerevisiae* strains, viz., Sc01, Sc02, Sc05, Sc12, Sc21, and Sc24 was prepared by inoculating 2% of seed inoculum to pasteurized apple juice and incubated at 28°C for 24 h under shaking conditions. Pasteurized apple juice was inoculated by 1% inoculum supplemented with di-Ammonium hydrogen phosphate (DAHP) (300 mg w/v) and incubated at room temperature for fermentation. The periodic samples were taken, spun at 6000 rpm for 5 min and analyzed for TSS, pH and ethanol content till no further decrease in °Brix was noticed. After completion of fermentation, analysis of the final product was carried out for various parameters, i.e., Estimation of pH, total soluble solids, titrable acidity (Amerine et al., 1967), brix-acid ratio, ethanol content (Caputi et al., 1968), ascorbic acid content (Ranganna, 1976), reducing sugars (Miller, 1950), and total sugars (Dubois et al., 1956).

### Sensory Evaluation

The organoleptic evaluation of cider was done on the basis of appearance, color, flavor, mouthfeel and overall acceptability by a panel of five judges. Consumer acceptance for the products was evaluated on a nine point “Hedonic scale” (Amerine et al., 1965).

### Statistical Analysis

All experiments were performed in triplicate and the results were analyzed statistically by one-way ANOVA and are presented as mean values with the standard error calculated at the 95% confidence level.

## Results and Discussion

### *ATF1* Gene Studies

During fermentation processes, yeast cells produce a broad range of aroma-active substances which greatly affect the complex flavor of fermented alcoholic beverages. While these secondary metabolites are often formed only in trace amounts, their concentrations determine the distinct aroma of these beverages. Flavor-active substances produced by fermenting yeast cells can be divided into five main groups: sulfur-containing molecules, organic acids, higher alcohols, carbonyl compounds, and volatile esters (Nykanen and Suomalainen, 1983; Nykanen, 1986; Hammond, 1993; Lambrechts and Pretorius, 2000; Pisarnitskii, 2001). Of these, volatile esters represent the largest and most important group. They are responsible for the highly desired fruity character of beer and, to a lesser extent, other alcoholic beverages, such as wine. The major flavor-active esters in beer are acetate esters such as ethyl acetate (solvent-like aroma), isoamyl acetate (banana flavor), and phenylethyl acetate (flowery, rose aroma). In addition, C_6_–C_10_ medium-chain fatty acid ethyl esters such as ethyl hexanoate (ethyl caproate) and ethyl octanoate (ethyl caprylate), which have “sour apple” aromas, are also important for the overall bouquet (Meilgaard, 2001).

The means of controlling ester synthesis during industrial beer fermentations are very limited (Verstrepen et al., 2001). It is well known that ester formation is highly dependent on the yeast strain used (Peddie, 1990) and on certain fermentation parameters. Alvarez et al. (1994) found a clear correlation between the concentrations of ethyl acetate and isoamyl acetate in beer, indicating that these esters may be synthesized by the same rate-limiting enzyme. The best-known enzymes involved in ester synthesis are the so-called alcohol acetyltransferases (AATases; EC 2.3.1.84), encoded by *ATF* genes (*ATF1, ATF2*, and *Lg-ATF1*). These enzymes catalyze the formation of acetate esters from the two substrates: alcohol and acetyl-CoA. It was shown that during fermentation, acetate ester production rates followed a pattern corresponding to the AATase activity (Malcorps et al., 1991). In one of the studies, overexpression of *ATF1* derived from an industrial lager brewer’s yeast strain resulted in a 27-fold increase in isoamyl acetate production and a 9-fold increase in ethyl acetate production compared to empty-vector transformants (Fujii et al., 1994). These studies indicate that the expression level of *ATF1* is an important limiting factor for ester synthesis under industrial conditions.

**FIGURE 1:**
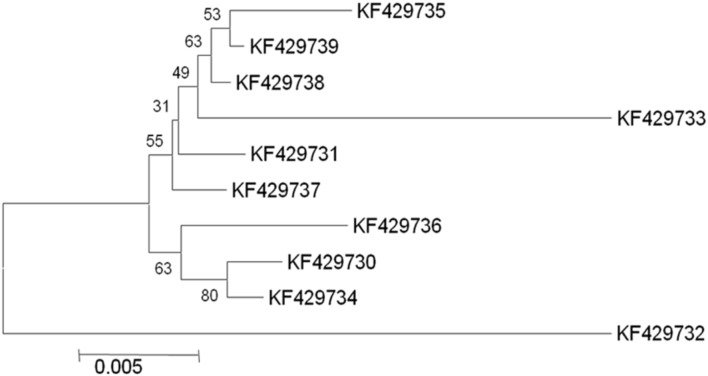
**Phylogenetic tree depicting variation in *ATF1* gene sequences with a scale of 0.005 substitutions per nucleotide position**.

**FIGURE 2:**
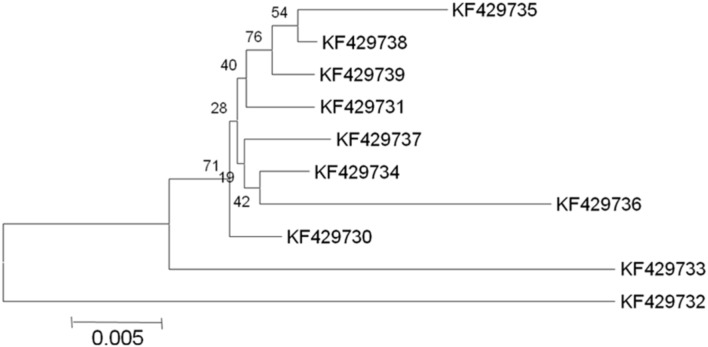
**Phylogenetic tree depicting variation in *ATF1* gene sequences with a scale of 0.005 substitutions per amino acid position**.

In selected *S. cerevisiae* strains, the *ATF1* gene was found to consist of 1566 bp open reading frame that encodes 522 amino acids. These results showed discrepancy from the earlier study reporting 1578 bp open reading frame of the structural gene encoding 525 amino acids in *S. cerevisiae* (Fujii et al., 1994). The sequences of the protein coding regions of *ATF1* gene showed a wide variation within these ten indigenous strains. Multiple sequence alignments revealed about 103 nucleotides substitutions at different locations without any deletions or insertions. Subsequent analysis of amino acid sequences of the *ATF1* genes revealed difference of about 47 amino acids among the indigenous yeast strains, suggesting great variations in aroma and flavor of the brewing products. Verstrepen et al. (2003) also showed that overexpression of different alleles of *ATF1* and *ATF2* leads to different ester production rates, indicating differences in the aroma profiles of yeast strains which may be partially due to mutations in their *ATF* genes. In phylogenetic trees (**Figures 1** and **2**) based on nucleotide and amino acid sequence analysis, the *ATF1* sequence of a strain, KF429732 (Sc01), was found to be highly dissimilar to other strains used in the study. This strain also had most desired organoleptic properties as evident from studies conducted with hard apple cider (**Table 5**). The phylogenetic tree obtained after amino acid sequence analysis of the *ATF1* gene (**Figure 2**) was almost similar to that obtained after analysis of nucleotide sequences. As evident from the results, *ATF1* gene can be used to reveal differences in ester formation among these indigenous yeast strains at genetic level.

### Organoleptic Studies

Cider is one of the most popular drinks all around the world. In apple producing countries, the apple crop and its subsequent transformation in order to obtain derivatives (brandy, vinegar, apple juice, etc.), is of enormous commercial, economic as well as social relevance. Many different strains of yeast and methods of fermentation are used for producing cider. The interest for locally produced food is increasing due to consumer concern about the environment, distrust of industrial foods and a demand for high quality products. Apple is the predominant fruit crop of Himachal Pradesh and processing of apples into cider could significantly contribute towards the development of the market. The choice of yeast strain as starter culture can have a high impact on the flavor profile of fermented beverages (Nurgel et al., 2009). During fermentation of apple juice, the rate and content of ethanol, sugars, tannins, esters, methanol, and volatile acids are some of the quality characteristics that can be affected by the specific yeast strain (Joshi et al., 2002). The physicochemical analysis of apple juice was evaluated on the basis of chemical analysis and is presented in **Table 2**.

**Table 2:** Physicochemical characteristics of apple juice.

Parameters	Juice
TSS (°Brix)	9.7
pH	3.617
Reducing sugars (mg/100 mL)	151.2
Total sugars (mg/100 mL)	276.7
Titrable acidity (%)	0.48
Asorbic acid (mg/100 g)	10.256
Brix:acid ratio	20.208

The fermentation conditions such as initial sugar concentration and temperature have been found to exert both positive and negative influence on the quality of beverage. The interaction between temperature and sugar concentration can determine the final quality of the beverage (Llaurado et al., 2002). Hence the sugar level of the pulp was adjusted to 18 °Brix using granulated sucrose. The pulp was inoculated with 1% of six selected yeast strains (Sc01, Sc02, Sc05, Sc12, Sc21, and Sc24) to evaluate the differences in their fermentation behavior. The samples were incubated at room temperature (25°C). Time course study of fermentation revealed 15 days optimum for hard cider preparation and 3 days for soft cider. The significant changes and differences up to 13 days were reported during fermentation of hard cider for every strain. Most of the parameters showed significantly different values after 15 days of fermentation (**Table 3**).

**Table 3:** Comparative physicochemical analysis of apple cider prepared by six *S. cerevisiae* strains after 15 days.

Strains	TSS(°Brix)	Alcohol content (%)	pH	Reducing sugars (mg/100 mL)	Total sugars (mg/100 mL)	Titrable acidity (%)	Ascorbic acid (mg/100 mL)	Brix:acid ratio	Log (CFU/mL)
Juice	18^a^	0.01^f^	3.617^a^	263.783^a^	660.743^a^	0.2633^e^	4.664^a^	68.367^a^	4.927^e^
Sc01	7^b^	5.433^b^	3.133^de^	126.193^b^	231.873^b^	0.4587^d^	1.287^b^	15.262^b^	8.793^a^
Sc03	5.767^c^	4.633^d^	3.143^de^	94.579^c^	195.013^c^	0.507^b^	0.641^c^	11.98^c^	8.623^b^
Sc05	5.4^d^	5.433^b^	3.41^b^	70.407^f^	142.006^d^	0.673^a^	0.642^c^	11.618^d^	8.773^a^
Sc12	5.63^cd^	4.1^e^	3.243^d^	79.647^e^	153.587^e^	0.5047^b^	0.6413^c^	11.374^c^	8.487^c^
Sc21	5.567^cd^	5.8^a^	3.037^e^	81.083^e^	158.483^f^	0.482^c^	0.639^c^	11.163^c^	8.56^b^
Sc24	5.766^c^	5.03^c^	3.35^c^	85.38^d^	173.867^g^	0.4813^c^	0.6413^c^	8.024^d^	8.333^d^
CD (5%)	0.2506	0.2228	0.1105	2.4986	4.3467	0.0065	0.07	1.0623	0.04201

*Results are shown as mean of three replications, different letters denote significant differences among values of various traits (P < 0.05).*

The apple cider samples were put to sensory analysis to find out the acceptability among the tasters. The soft and hard apple cider was subjected to evaluation by a panel of five judges on a 9 point ‘Hedonic scale’. The soft cider prepared from Sc21 *S. cerevisiae* strain was found to be best among all other cider preparations (**Table 4**) and hard cider prepared by Sc01 strain was found to be of standard quality (**Table 5**) having 5.43% alcohol (v/v) and 7 °Brix of sugar.

**Table 4:** Sensory evaluation of soft cider prepared by using six *S. cerevisiae* strains.

Sr. No.	Sample code	Sensory parameters				
		Appearance/color	Flavor	Mouthfeel	Taste	Overall acceptability
1	Sc01	8	5	5	4	5.5
2	Sc03	7	7	6	5	6.25
3	Sc05	6	6	6	5	5.75
4	Sc12	8	8	8	8	8
5	Sc21	8	9	9	9	8.75
6	Sc24	5	6	6	6	5.75

**Table 5:** Sensory evaluation of hard cider prepared by using six *S. cerevisiae* strains.

Sr. No.	Sample code	Sensory parameters				
		Appearance/color	Flavor	Mouthfeel	Taste	Overall acceptability
1	Sc01	8	8	8	8	8
2	Sc03	8	7	6	6	6
3	Sc05	7.5	5	5	5.2	5.8
4	Sc12	8	5	5	6	4
5	Sc21	8	6	6	6	6
6	Sc24	7	7.5	7	7	7

## Conclusion

*ATF1* gene studies revealed wide variation within the 10 indigenous yeast strains, suggesting great variation in aroma and flavor of the brewing products. These findings signify that this gene can play role in revealing the differences in ester formation among indigenous *S. cerevisiae* strains. However, other gene groups associated with this trait are further needed to be studied as they are also important factors in deciding the aroma and flavor of brewing products. The *ATF1* gene sequence of Sc01 was found to be dissimilar to other strains used in the study and the organoleptic properties of this strain were most desirable among all the indigenous yeast strains. Sensory analysis suggested Sc21 and Sc01 as best strains for soft and hard apple cider, respectively, indicating their role in enhancing the quality of apple products.

## Author Contributions

All authors listed, have made substantial, direct and intellectual contribution to the work, and approved it for publication.

## Conflict of Interest Statement

The authors declare that the research was conducted in the absence of any commercial or financial relationships that could be construed as a potential conflict of interest.
